# Bioassay Directed Isolation, Biological Evaluation and in Silico Studies of New Isolates from *Pteris cretica* L.

**DOI:** 10.3390/antiox8070231

**Published:** 2019-07-19

**Authors:** Farooq Saleem, Rashad Mehmood, Saima Mehar, Muhammad Tahir Javed Khan, Zaheer-ud-Din Khan, Muhammad Ashraf, Muhammad Sajjad Ali, Iskandar Abdullah, Matheus Froeyen, Muhammad Usman Mirza, Sarfraz Ahmad

**Affiliations:** 1 Punjab University College of Pharmacy, University of the Punjab, Lahore 54000, Pakistan; 2 Faculty of Pharmacy, University of Central Punjab, Lahore 54000, Pakistan; 3 Department of Chemistry, University of Education, Vehari Campus, Vehari 61100, Pakistan; 4 Department of Chemistry, Sardar Bahadur Khan Women University, Quetta 87300, Pakistan; 5 Botany Department, Government College University, Lahore 54000, Pakistan; 6 Department of Pharmacy, The Islamia University of Bahawalpur, Bahawalpur 63100, Pakistan; 7 Institute of Molecular Biology and Biotechnology, University of Lahore, Lahore 54600, Pakistan; 8 Department of Chemistry, Faculty of Science, University of Malaya, Kuala Lumpur 50603, Malaysia; 9 Department of Pharmaceutical and Pharmacological Sciences, Rega Institute for Medical Research, Medicinal Chemistry, University of Leuven, B-3000 Leuven, Belgium

**Keywords:** *Pteris cretica* L., isolation, molecular docking, neurodegenerative, α-glucosidase, 2,2-diphenyl-1-picrylhydrazyl (DPPH)

## Abstract

Members of genus *Pteris* have their established role in the traditional herbal medicine system. In the pursuit to identify its biologically active constituents, the specie *Pteris cretica* L. (*P. cretica*) was selected for the bioassay-guided isolation. Two new maleates (F9 and CB18) were identified from the chloroform extract and the structures of the isolates were elucidated through their spectroscopic data. The putative targets, that potentially interact with both of these isolates, were identified through reverse docking by using in silico tools PharmMapper and ReverseScreen3D. On the basis of reverse docking results, both isolates were screened for their antioxidant, acetylcholinesterase (AChE) inhibition, α-glucosidase (GluE) inhibition and antibacterial activities. Both isolates depicted moderate potential for the selected activities. Furthermore, docking studies of both isolates were also studied to investigate the binding mode with respective targets followed by molecular dynamics simulations and binding free energies. Thereby, the current study embodies the poly-pharmacological potential of *P. cretica*.

## 1. Introduction

In the history of human civilization, plants have established a role in treating various ailments thereby reducing morbidity and mortality. With the dawn of modern chromatographic and spectroscopic techniques, this rich source of bioactive compounds was and is being extensively studied for the pursuit of high-end medicines. The small-molecule natural products are secondary metabolites of plants biosynthesized mainly as a defense mechanism against pathogenic microorganisms, insects and herbivores [1,2]. From 1981 to 2014, among small-molecule drugs approved by the FDA, 6% were unaltered natural products, 26% natural product derivatives and 27% synthetic drugs which were either a mimic of natural products or have natural product pharmacophores [3].

The genus *Pteris* (*Pteridaceae*) comprises of about 250 species which are widely distributed on all continents excluding Antarctica, with the highest diversity in the tropical and subtropical area, particularly including New Zealand, Australia, South Africa, Japan, America, China, and Europe [4]. Various species of *Pteris* have long been used for the treatment of different human ailments. *Pteris cretica* L. (*P. cretica*) is among the most commonly used species of *Pteris* in Chinese traditional medicines as an antidote, antipyretic, in burn treatment, antimicrobial and wound healing [5]. Sifting of literature shows that sesquiterpenes, ent-kaurane diterpenes and flavonoids have so far been reported from this plant [6]. We have previously reported the antimicrobial and antioxidant potential of this plant [7]. The current study is an extension of our previously published work. Herein, we report the bioassay-directed isolation and identification of two new isolates (**F9** and **CB18**) from two bioactive subfractions of the chloroform extract of *P. cretica,* along with their structure elucidation, antioxidant, antimicrobial, acetylcholinesterase (AChE) inhibition and α-glucosidase inhibition potentials. The screening of potential molecular targets through reverse in silico studies and the stability and binding free energies are also reported here. The results show that the isolates have moderate potentials for all tested biological activities.

Reactive oxygen species (ROS) produced in our normal metabolic pathway are believed to be responsible for various life-threatening disorders such as cardiovascular disorders, cancer, stress, atherosclerosis, Alzheimer’s, Parkinson’s, etc. [8,9]. The compounds capable of devouring ROS are termed as antioxidants and could be used as a defense against these life-threatening disorders. Alzheimer’s is one of the chronic neurodegenerative diseases characterized by loss of memory, mental abnormality and behavioral instabilities [10,11]. Its prognosis involves the upregulation of acetylcholinesterase (AChE) in the brain cell resulting in diminished amounts of acetylcholine neurotransmitter, which in turn alter smooth brain functions including memory loss. This disorder could possibly be controlled by inhibiting the AChE enzyme, and the candidates capable of this property are called AChE inhibitors. Tacrine, donepezil, rivastigmine and galantamine are commonly used as AChE inhibitors. 

The overall scheme for discovering molecular targets of phytochemicals have been uplifted by a variety of in silico tools such as the computer-aided drug discovery [12,13,14,15,16], combinatorial chemistry [17,18,19] and high throughput screening [20,21,22,23,24,25,26]. After identifying lead compounds, directly interacting potential targets are identified using the inverse/reverse docking methodology [27,28] on known crystal structures or using various proteomic tools [29,30]. The target identification approaches involving proteomics profiling and pharmacokinetics studies are laborious and time consuming [31]. Therefore, in silico-based target identification methods are always found as the efficient alternative [32]. Within in silico-based target identification of phytochemicals, various tools are used such as computer-aided drug discovery [13,14], combinatorial chemistry [17,18], high throughput screening [25,26] and large-scale reverse pharmacophore mapping strategy. Among various target prediction tools, the large-scale reverse pharmacophore mapping strategy for potential drug targets identification has appeared to be a potential optional technique [28]. The principle of reverse pharmacophore mapping involves the placement of flexible conformation of every small molecule into every pharmacophore model of proteins in the target list [33]. Presently, various in silico tools have been developed that use different target fishing algorithms to execute reverse docking. Few widely used tools include, PharmMapper [33], ReverseScreen3D [34], INVDOCK [35], Tarfisdock [36], idTarget [37], TargetHunter [38]. By utilizing these in silico tools, various studies have been published, including the identification of molecular targets (i.e., proapoptotic, anti-proliferative, anti-inflammatory, anti-invasive, anti-angiogenic) for tea polyphenols [39], saffron bioactive agents [40], essential oils in cardamom [41] and azadirachtin (the principle bioactive compound of neem seeds) [42].

The present study aimed at the isolation and identification of bioactive constituents from *P. cretica*. The potential molecular targets were screened using the reverse docking procedure. To further investigate the binding pose, the molecular dynamic simulations protocol was employed to analyze the stability and binding free energies of docked complexes. Based on in silico outcomes, selected enzyme inhibition assays were performed along with antibacterial activities. The study revealed the poly-pharmacological potential of bioactive compounds in *P. cretica*, responsible for its ethnobotanical uses and biological activities.

## 2. Material and Methods

Column chromatography (CC) was performed on silica gel 60 (70–230 mesh, E. Merck, Darmstadt, Germany). The thin layer chromatography (TLC) was performed on pre-coated silica gel 60 F_254_ plates (E. Merck, Darmstadt, Germany) and the detection was done at 254 and 330 nm or by spraying ceric sulfate in 10% H_2_SO_4_ (heating). High performance liquid chromatography (HPLC) was performed on the Hitachi Primaide HPLC system (Hitachi, Tokyo, Japan) with C18 columns of Thermo Scientific Hypersil GOLD (5 µm, 175 Å, 4 × 250 mm, Waltham, MA, USA), while the large-scale purification was performed with Thermo Scientific Hypersil GOLD (5 µm, 175 Å, 21.2 × 250 mm). Melting points were measured on a Gallenkamp apparatus (Loughborough, England) and optical rotations were measured on a JASCO DIP-360 polarimeter (Jasco, Tokyo, Japan). UV spectra were recorded on a Hitachi UV-3200 spectrophotometer (Hitachi, Tokyo, Japan) while the FTIR spectra were recorded on a Shimadzu FTIR-8900 spectrometer (Shimadzu, Kyoto, Japan). The ^1^H- and ^13^C-NMR spectra were recorded on Bruker AM-400 and AM-500 spectrometers (Bruker BioSpin, Faellanden, Switzerland) in deuterated solvents. The 2D (COSY, HMQC, HMBC) NMR spectra were recorded on the same instruments. The chemical shifts are reported in ppm (*δ*), relative to the tetramethylsilane (TMS) as an internal standard and the scalar couplings are reported in Hertz (Hz). Mass spectra (MS and HR-MS) in the FAB (fast atom bombardment) mode on Jeol JMS HX 110 spectrometer (Jeol, Tokyo, Japan) were used with glycerol as matrix and ions were given in *m/z* (%). All reagents, solvents and chemicals used were of analytical grade and used without any further purification. 

### 2.1. Plant Collection and Identification

*P. cretica* whole plant was collected from Azad Kashmir, a state of Pakistan, in April 2013 and identified by Prof. Dr. Zaheer-ud-Din Khan, Department of Botany, Govt. College University, Lahore, Pakistan, where a voucher specimen has been deposited in the herbarium (voucher specimen no. is 2142).

### 2.2. Extraction and Isolation of Compounds

The whole plants material of *P. cretica* was shade dried (1 kg), ground to powder and extracted with chloroform in a Soxhlet apparatus to dark brown residue (34 g). The residue (34 g) was subjected to column chromatography (CC) over silica gel and eluted with hexane-acetone in order to increase polarity to collect 17 fractions. All the obtained fractions were subjected to measure their antioxidant activity and biological potential against five bacteria. The fractions obtained with 6.5:3.5 and 5.5:4.5 hexane-acetone mixtures were found most active against *S. aureus* and *E*. *coli* with 37.66%, 17.66% and 38.66%, 16.33% zones of inhibition respectively as compared to the control ampicillin (100% and 94.33%). The fraction obtained with hexane-acetone (1.8:1, 0.5 g) was further purified by CC over silica gel and eluted with dichloromethane (DCM)-methanol mixture in order to increase polarity to collect thirteen subfractions, and all subfractions were subjected to measure their biological activities. The most bioactive subfraction, which was obtained with DCM-methanol (19:1, 135 mg), was rechromatographed over silica gel and eluted with the same solvent system (DCM-methanol) to collect further several subfractions. The most biological active subfraction which was subjected to p-HPLC using water-acetonitrile= 45:55 to 100% acetonitrile to obtain the pure compound (**F9,** 5 mg). 

The second bioactive main fraction, which was obtained with hexane-acetone (1.2:1, 1.5 g) was subjected to CC over silica gel and eluted with DCM-methanol in increasing order of polarity to collect 12 subfractions. All the subfractions were subjected to biological activity, the subfraction which was obtained with DCM-methanol (5.7:1, 65 mg) was the most active subfraction, and subsequently this subfraction was subjected to p-HPLC using water-acetonitrile (2:8) to afford the pure compound (**CB18**, 6 mg).

#### 2.2.1. 2-Ethyloctyl Maleate (F9)

Colorless amorphous solid, UV λ_max_ (MeOH) nm (log ε): 285 (2.1). IR υ_max_ (KBr) cm^−1^: 3334 (hydroxyl), 2892 br. (carboxylic acid hydroxyl), 1730 (carbonyl), 1653 (conjugated olefin). ^1^H-NMR (CDCl_3_, 500 MHz) δ (ppm): 7.68 (1H, d, *J* = 9.0 Hz, H-3), 7.50 (1H, d, *J* = 9.0 Hz, H-2), 4.19 (2H, dd, *J* = 14.5, 6.0 Hz, H-1’), 1.65 (1H, m, H-2’), 1.39 (2H, m, H-1’’), 1.32 (2H, m, H-3’), 1.30 (2H, m, H-4’), 1.29 (4H, m, H-5’,-6’), 1.26 (2H, m, H-7’), 0.90 (3H, t, *J* = 7.5 Hz, H-2’’), 0.86 (3H, t, *J* = 7.5 Hz, H-8’), ^13^C-NMR (CDCl_3_, 150 MHz) δ (ppm): 167.8 (C-1), 130.9 (C-2), 128.8 (C-3), 167.8 (C-4), 68.1 (C-1’), 38.7 (C-2’), 30.3 (C-3’), 28.9 (C-4’), 29.4 (C-5’), 31.9 (C-6’), 23.0 (C-7’), 14.1 (C-8’), 23.7 (C-1’’), 11.0 (C-2’’). HR-FAB-MS *m/z* 257.1747 [M+H]^+^ (calcd. for C_14_H_25_O_4_, 257.1753).

#### 2.2.2. 2-Ethylhexyl Maleate (CB18)

Colorless amorphous solid, UV λ_max_ (MeOH) nm (log ε): 287 (2.2). IR υ_max_ (KBr) cm^−1^: 3335 (hydroxyl), 2898 br. (carboxylic acid hydroxyl), 1732 (carbonyl), 1655 (conjugated olefin). ^1^H-NMR (CD_3_OD, 400 MHz) δ (ppm): 7.72 (1H, d, *J* = 8.8 Hz, H-3), 7.62 (1H, d, *J* = 8.8 Hz, H-2), 4.22 (2H, dd, *J* = 5.6, 2.0 Hz, H-1’), 1.68 (1H, m, H-2’), 1.43 (2H, m, H-1’’), 1.36 (2H, m, H-3’), 1.34 (2H, m, H-4’), 1.32 (2H, m, H-5’), 0.94 (3H, t, *J* = 7.5 Hz, H-6’), 0.91 (3H, t, *J* = 7.2 Hz, H-2’’). ^13^C-NMR (CD_3_OD, 150 MHz) δ (ppm): 169.4 (C-1), 132.4 (C-2), 129.9 (C-3), 169.4 (C-4), 69.1 (C-1’), 40.2 (C-2’), 31.6 (C-3’), 30.1 (C-4’), 24.0 (C-5’), 14.4 (C-6’), 24.9 (C-1’’), 11.4 (C-2’’). HR-FAB-MS *m/z* 229.1430 [M+H]^+^ (calcd. for C_12_H_21_O_4_, 229.1440).

#### 2.2.3. In Silico Drug Likeness and ADMET Properties of Active Compounds in *P. cretica*

In order to check the drug-like potential of active compounds in *P. cretica*, physicochemical properties were determined from Mcule (https://mcule.com/) [43]. Drug-likeness and the drug score of the compounds, which is based on topological descriptors, fingerprints of molecular drug likeness, structural keys or other properties as *logP* and molecular weights were determined from the Molinspiration program (http://www.molinspiration.com/cgi-bin/properties) and Osiris Property Explorer (http://www.organic-chemistry.org/). Detailed ADMET (Absorption, Distribution, Metabolism, Excretion and Toxicity) were predicted from the admetSAR webserver (http://lmmd.ecust.edu.cn:8000/predict/) [44].

### 2.3. Identification of Putative Therapeutic Target

To find the potential therapeutic targets for active compounds of *P. cretica,* dual reverse screening approaches were employed by using the ReverseScreen 3D [34] and PharmMapper webservers [33]. ReverseScreen 3D screens the potential targets against biological active small compounds from the updated subsets of co-crystallized ligands present in the Protein Data Bank (PDB). PharmMapper is another reverse screening webserver, which employs pharmacophore-mapping strategy for the identification of potential targets for given small compounds. PharmMapper carries a large, internal database namely PharmTargetDB annotated from all the target information in BindingDB [45], DrugBank [46] and the potential drug target database (PDTD) [47]. Active compounds from *P. cretica* were submitted to the ReverseScreen 3D and PharmMapper in the default format. The list of potential targets was further investigated to filter out the targets associated to anticancer, antioxidant, anti-inflammatory, and neurodegenerative mechanisms. The high throughput experimental findings of potential targets were manually curated from three different bioassay databases: PubChem Bioassay [48], NPACT [49] and Herbal Ingredients’ Targets Database [50]. The list of potential targets identified by the in silico reverse screening method was compared with the target information acquired from three bioassay databases as an experimental evidence. Based on the results, various identified targets such as anti-proliferative, antioxidant and anti-inflammatory targets were sorted out. Alongside the reverse docking, the antibacterial efficacy of the identified compounds was also evaluated by carefully taking into account the potential antibacterial targets after literature review.

### 2.4. Experimental Protocols for Biological Assays

To validate the therapeutic targets identified by the in silico reverse docking procedure, various biochemical assays were performed. 

### 2.5. Antibacterial Assay

The antibacterial potential of isolated compounds **F9** and **CB18** was measured against Gram-negative *Escherichia coli* (*E. coli*), *Shigella flexneri* (*S. flexneri*), *Pseudomonas aeruginosa* (*P. aeruginosa*) and *Klebsiella pneumoniae* (*K. pneumoniae*), and Gram-positive *Staphylococcus aureus* (*S. aureus*) bacteria by using a Microplate Alamar Blue Assay (MABA) [51]. The selected bacteria were taken from the Pakistan Council of Scientific and Industrial Research (PCSIR) laboratory, Lahore, Pakistan. Muller Hinton media (MHM) was used for bacterial culturing and 0.5 McFarland turbidity index was maintained for inoculums. MHM (50 µL) was inoculated to each well of 96-well microtiter plate under sterile conditions. A volume of 10 µl from each test sample was pipetted into all labelled wells excluding control wells. Each well was added with MHM to make a final volume of 200 µL. A specific bacterial suspension with a volume of 7 µL (5 × 10^6^ cfu/mL) was added to all wells in the plate. To avoid a dehydration issue in bacteria, parafilm was used to cover the microplate and then placed for incubation at 35 °C for 18 to 20 h. At this phase, the microplate contained a set of samples, standard and a negative control. Next to the incubation period, a multichannel pipette was used to pipette 10% resazurin (dye) to total culture volume into each well. Now the microplate was mixed with a speed of 80 rpm in a shaking incubator for 2 to 3 h. During the blending process, it was ensured that the parafilm remained attached with the plates. The change in color from blue to pink was the assessment of bacterial growth (viability).

The diminution in blue color concentration was measured with the ELISA reader at 570 nm and 600 nm wavelengths. The percentage inhibition was calculated with the following formula and repeated thrice to find the mean inhibition and standard deviation.

$$\%\;\mathrm{Difference}=\frac{\left(\varepsilon_{\mathrm{ox}}\right)\;\lambda_{2}A\;\lambda_{1}-\left(\varepsilon_{\mathrm{ox}}\right)\;\lambda_{1}A\;\lambda_{2}\;\mathrm{of}\;\mathrm{test}\;\mathrm{agent}\;\mathrm{dilution}}{\left(\varepsilon_{\mathrm{ox}}\right)\;\lambda_{1}Å\;\lambda_{2}-\left(\varepsilon_{\mathrm{ox}}\right)\;\lambda_{2}Å\lambda_{1}\;\mathrm{of}\;\mathrm{untreated}\;\mathrm{positive}\;\mathrm{growth}\;\mathrm{control}}\times 100$$

where, ε_ox_ is the molar extinction coefficient of Alamar blue oxidized form (blue), A is the absorbance of the test well, Å is the absorbance of the positive growth control well, λ_1_ is the wavelength at 570 nm and λ_2_ is the wavelength at 600 nm.

### 2.6. Antioxidant Activity 

Stock solutions of test compounds **F9** and **CB18** and standard vitamin C were prepared in ethanol at a concentration of (1 mg/mL). Each well of 96-well microtiter plate was loaded with 50 µL of ethanol. The serial dilutions (128, 64, 32, 16, 8, 4, 2, 1, 0.5, 0.25, 0.125 µg/mL) were prepared by using 50 µL of stock solution. The ethanolic solution of DPPH (150 µL, 0.05 mM) was added to each well. Control wells contained 50 µL ethanol and 150 µL DPPH solution. The plate was incubated for 30 min in the dark and the absorbance was read at 517 nm. The antioxidant activity was expressed as the percentage radical scavenging activity (%RSA) and calculated by using the following expression.

%RSA = (Ac − As)/Ac × 100

where, Ac and As are the absorbance of control and sample, respectively. The experiment was repeated thrice to find the mean %RSA ± standard deviation and *IC_50_* was calculated by plotting the log of concentration against the mean %RSA.

### 2.7. Acetylcholinesterase (AChE) Inhibition Activity

The AChE inhibition potential of isolates was investigated according to the reported procedure with slight modifications [52]. The methanolic solution of the test compound or standard (Eserine) (10 µL, 0.5 mM) was added to 96-well plates containing 60 µL phosphate buffer (pH = 7.7, 50 mM). Control wells contained 60 µL phosphate buffer and 10 µL methanol. This was followed by loading 10 µL enzyme (0.005 units for AChE) to each well. The wells contents were shaken and the absorbance at 405 nm was measured. The plate was incubated at 37 °C for 10 min and the substrate (acetylthiocholine iodide) (10 µL, 0.5 mM) and 5, 5-Dithio-bis-(2-Nitrobenzoic Acid) (10 µL, 0.5 mM) were added to each well. The plate was incubated for 15 min at 37 °C and the absorbance was measured at 405 nm using a 96-well plate reader. The test was repeated in triplicate and the percentage inhibition was calculated by following the formula.

% Inhibition = (Ac − As)/Ac × 100

### 2.8. α-Glucosidase (GluE) Inhibition Assay

α-Glucosidase (GluE) inhibition assay was carried out with slight modifications in the method performed by Chapdelaine [53]. The wells of the 96-well microtiter plate were loaded with 70 µL phosphate buffer saline (50 mM, pH = 6.8) and the test samples or Acarbose as standard (10 µL, 0.5 mM). The control wells contained 70 µL phosphate buffer and 10 µL methanol. A volume of 10 µL GluE enzyme (0.057 units) was pipetted into each well followed by proper mixing. After 15 min of pre-incubation period (at 37 °C), the absorbance was recorded at 400 nm. For the initiation of reaction, 10 µL of p-nitrophenyl glucopyranoside (0.5 mM) was added as the substrate. The plate was incubated for 30 min at 37 °C before measuring the absorbance at 400 nm. The percentage inhibition was calculated by the same following formula and the experiment was repeated in triplicate to find the mean and standard deviation.

% Inhibition = (Ac − As)/Ac × 100

### 2.9. Molecular Docking of Potential Targets with Bioactive Compounds

To investigate the binding mode and molecular interactions of investigated compounds with therapeutic targets as identified with the experimental procedure, molecular docking studies was employed using Autodock Vina with default setting and parameters [54]. The X-ray determined crystallographic 3D structures of identified potential targets including AChE (PDB ID: 4BDT), based on the mechanism involved in neurodegeneration were retrieved from PDB with a good resolution and R-free factor [55] while *Saccharomyces cerevisiae* α-glucosidase enzyme was modeled using *Saccharomyces cerevisiae* isomaltase (PDB ID: 3AJ7, Identity: 72.4%) as a template due to the unavailability of crystal structure. SWISS-MODEL [56] was utilized for homology modeling and the model was refined through 20 ns MD simulations. For antimicrobial targets, an extensive literature review was performed and the dihydropteroate synthase (DHPS) from *Escherichia coli*, and dihydrofolate reductase (DHFR) from *Staphylococcus aureus* were selected for docking studies and there the corresponding PDB IDs were 5U10 (*E. coli* DHPS complex with pteroic acid) [57] and 4LAE (*S. aureus* DHFR complex with 7-(Benzimidazol-1-yl)-2,4-diaminoquinazolines) (PDB ID: 4LAE) [58]. 

For the structure preparation, co-crystalized ligands were separated from the corresponding proteins and re-docked again to calculate the RMSD of docked and co-crystalized ligand for reliability of the AutoDock Vina protocol [54]. The overall protocol of the protein preparation, minimization and optimization has been distinctively described elsewhere [59,60,61]. The molecular structures of potent bioactive compounds **F9** and **CB18** were generated from the ACD/ChemSketch Freeware, the software was employed to design the 2D structure and OpenBabel was used to convert 2D to 3D structure. AutoDock Tools were used to merge non-polar hydrogens, add Gasteiger charges and rotatable bonds adjustments. The docked complexes were investigated for molecular interactions utilizing UCSF Chimera v10 [62].

### 2.10. Molecular Dynamics Simulations and Binding Free Energy Calculations

To understand the dynamic behavior with respect to time (nanoseconds, ns), the best-docked complexes from molecular docking studies were executed through 20 ns MD simulations. All MD simulations were executed using the AMBER 16 package [63]. We implemented the same MD simulation protocol as described in our previous study [64]. The CPPTRAJ module of AMBER 16 [65] was used for the trajectory analysis.

To further explore the binding free energy calculations between selected proteins with bioactive compounds separately. A total of 500 snapshots were extracted from the whole trajectory for binding free energy calculations using the molecular mechanics-generalized Born surface area (MMGBSA) method. MMGBSA calculations were performed using the mmpbsa module of AMBER 16 package. The details of the MMGBSA method have been explained elsewhere [66,67]. The MMGBSA total energy (ΔG_tol_) was divided into molecular mechanics (ΔE_MM_) and solvation energy (ΔG_sol_) contributions. ΔE_MM_ is further divided into electrostatic energy contributions (ΔE_ele_) and van der Waals (ΔE_vdw_) while the solvation energy is divided into polar (ΔG_p_) and nonpolar (ΔG_np_) contributions.

$$\begin{array}{c} \Delta E_{\mathrm{MM}}=\Delta E_{\mathrm{int}}+\Delta E_{\mathrm{ele}}+\Delta E_{\mathrm{vdw}}\\ \Delta G_{\mathrm{sol}}=\Delta G_{p}+\Delta G_{\mathrm{np}}\\ \Delta G_{\mathrm{tol}}=\Delta E_{\mathrm{MM}}+\Delta G_{\mathrm{sol}} \end{array}$$

## 3. Results and Discussion

In our previous study on *P. cretica*, n-hexane, chloroform and ethanol extracts of *the* whole plant were screened for antimicrobial and antioxidant activities. The chloroform extract was found the most active among all studied extracts [7]. The current study was commenced to isolate the active ingredients from the chloroform extract. The shade dried whole plant was ground and extracted with chloroform using the Soxhlet extractor. The crude extract was subjected to repeated column chromatography steps, each followed by biological activates of fractions in a bioassay-guided manner to identify the most active fractions. After column chromatography steps, most active fractions were subjected to the reverse phase HPLC to isolate the pure ingredients. 

Compound **F9** was obtained as a white amorphous powder. The UV spectrum showed the absorption bands at 285 nm, while the IR spectrum exhibited absorptions for the presence of hydroxyl (3334 cm^−1^), carboxylic acid hydroxyl (2892 br. cm^−1^), carbonyl (1732 cm^−1^), and olefinic (1655 cm^−1^) moieties. The molecular formula was deduced as C_14_H_25_O_4_ by high resolution FAB-MS (fast atom bombardment-mass spectrum), which showed the pseudo molecular ion [M+H]^+^ peak at *m/z* 257.1747. Two downfield doublets of ^1^H-NMR spectrum of **F9** (Table 1) of olefinic protons at δ 7.68 and 7.50 with coupling constant 9.0 Hz revealed the presence of olefinic bond having *cis* geometry. A methylene proton signal at δ 4.19 (dd, *J* = 14.5, 6.0 Hz) showed its connectivity with oxygen and methine multiplet at δ 1.65 (1H, m) in COSY. The high frequency region was a characteristic of hydrocarbon chain methylenes within the range of δ 1.29–139 (12H) and the presence of two terminal methyl groups was revealed by two triplets at *δ* 0.90 (t, *J* = 7.5 Hz) and 0.86 (t, *J* = 7.5 Hz), which indicated the presence of a branch in the hydrocarbon chain. ^13^C-NMR (BB and DEPT) spectra showed the carbonyl carbons signal at δ 167.8 while the olefinic carbons showed signals at δ 130.9 and 128.9. The oxygenated methylene carbon was observed at δ 68.1 whereas a methine carbon showed the signal at δ 38.1. The methylene carbons of the hydrocarbon chain were resonated in the range of δ 23.0–31.9 with the two terminal methyl signals at δ 14.1 and 11.0.

**Figure 1:**
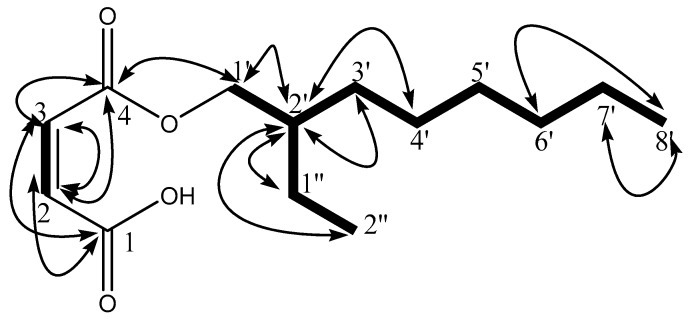
Structure of compound **F9** (2-ethyloctyl fumarate) with important ^1^H-^1^H COSY (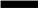) and ^1^H-^13^C HMBC (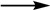) correlations.

In COSY correlations of F9 (Figure 1), the proton at δ 4.19 (H-1’) showed the COSY correlation with the proton at δ 1.65 (H-2’), which subsequently showed a correlation with the proton at δ 1.39 (H-1”) and δ 1.32 (H-3’), revealing the presence of the branch at C-2’. In the HMBC experiment (Figure 1), olefinic protons at δ 7.68 and 7.50 showed ^2^*J* and ^3^*J* correlations with the carbonyl at δ 167.8, confirming the presence of olefinic bond adjacent and in conjugation with the carbonyls. The proton at δ 4.19 (H-1’) showed the ^3^*J* correlations with carbonyl carbon at δ 167.8 (C-4), which confirms the presence of ester moiety and connectivity of C-1’ with ester oxygen. The proton at δ 4.19 (H-1’) also showed ^3^*J* with δ 30.3 (C-3’) and 23.7 (C-1”), and ^2^*J* correlation at δ 38.1 (C-2’), and subsequently H-2’ (δ 1.65) showed key ^2^*J* correlations at δ 30.3 (C-3’) and 23.7 (C-1”) and ^3^*J* correlation at δ and 11.0 (C-2”), confirming the branch is ethyl group and its connectivity at C-2’. The remaining HMBC correlations illustrated in Figure 1 along with COSY correlations were in complete agreement with the assigned structure of compound **F9** as 2-ethyloctyl maleate (Figure 1). 

**Figure 2:**
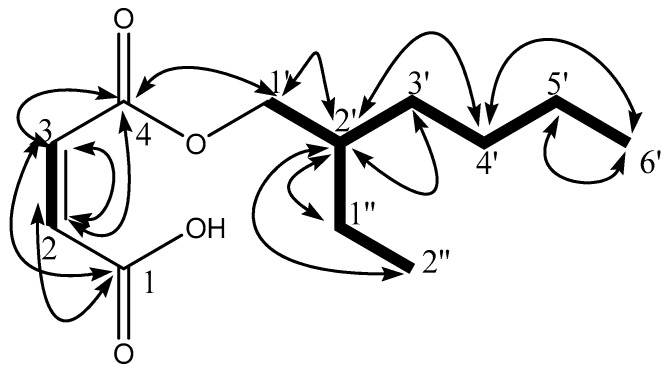
Structure of compound **CB18** (2-ethylhexyl fumarate) with important ^1^H-^1^H COSY (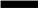) and ^1^H-^13^C HMBC (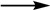) correlations.

Compound **CB18** was also obtained as a white amorphous powder. The UV and IR spectra were similar to those of compound **F9**. HR-FAB-MS showed the pseudo molecular ion [M+H]^+^ peak at *m/z* 229.1433 consistent to the molecular formula C_12_H_21_O_4_. Compound **CB18**, therefore, differed from compound **F9** in having two methylene groups less in the hydrocarbon chain, as [M+H]^+^ peak of compound **CB18** was *m/z* 28 less to that of compound **F9**. The ^1^H and ^13^C NMR spectra showed common features resemblance to those of compound **F9**, allowing to assign the structure of compound **CB18** as 2-ethylhexyl maleate (Figure 2).

### 3.1. Putative Therapeutic Target for Isolates

The list of potential top-ranked targets predicted from PharmMapper and ReverseScreen3D were tabulated in Table 1. Most of these targets are associated to anticancer, anti-inflammatory and neurodegenerative mechanisms as demonstrated in proceeding sections. The pharmacokinetics/ADMET estimations of both bioactive compounds are tabulated in Supplementary Table S1. 

### 3.2. Antiproliferative Targets

The process of growth and differentiation is under tight regulation, controlled by growth factors and their receptors. Abnormal growth and development may result as a consequence of slight variations in the regulation of expression of the molecules associated, causing malignant transformation. An increase in the expression level of growth factors for instance, transforming growth factor-α (TGF-α), can lead to non-cancerous disease, one such example is psoriasis. Anticancer studies have demonstrated a significant role of growth factors regarding anticancer activity [68]. Proliferation of a cancerous cell has portrayed an over-expression of the epidermal growth factor receptor (EGFR) as a prominent factor [68,69,70]. Two distinct signaling pathways regulate the apoptotic process which forms a loop-structure after their merger as a result of the effector molecule, Caspase 3 activation [71]. Reverse Screening has helped us to figure out various growth factors that can act as potential targets for bioactive compounds identified in *P. cretica*. The basic fibroblast growth factor receptor 1 and placental growth, epidermal growth factor receptor and VEGFR2 were estimated to bind with both compounds. The anti-angiogenesis potential of *P. cretica* might be central to the binding to these growth factors. The proapoptotic activity has been linked with these bioactive compounds, which bind to BCL-XL, Caspase 3 and Caspase 7 for further activation. Cyclins play a key role in different phases of a cell cycle. Three types of cyclins (cyclin D1, cyclin D2, cyclin D3) bind to CDK4 and to CDK6 and CDK-cyclin D complexes to regulate the entry in the G1 phase [72,73]. This study identified that the proliferation inhibition can be brought about by targeting Cyclin-A2 and cyclin dependent kinases 2 causing G1 cell cycle arrest through **CB18** and **F9**. This process promotes apoptotic cell death and hence, estimated the onco-protective potential of *P. cretica*. As tabulated in Table 1, a set of other targets including MAP kinase 14, Estrogen receptor Beta, p53, revealed **CB18** and **F9** of significant importance with respect to cancer-preventive potential of *P. cretica*.

### 3.3. Antioxidant and Antiinflammatory Targets

Studies centered upon carcinogenesis have indicated inflammation to be of prime importance in the process, involving tumor initiation, promotion and its progression. Acute inflammation though observed to play a significant role in defense response, cancer has been found to be caused by chronic inflammation [74]. For their role in the inhibition of proliferation, angiogenesis, invasion and metastasis, pro-inflammatory gene products have been in the spotlight over the last two decades [75]. Studies have demonstrated that the progression of various forms of cancer involves key components of inflammatory pathways such as cytokines, signal transducer and activator of transcription-3 (STAT-3), prostaglandins, free radicals, vascular endothelial growth factor (VEGF), NF-κB, cyclooxygenase-2 (COX-2) and inducible nitric oxide synthase (iNOS) [76,77]. In the progression of inflammation, in controlling the factors that deal with the immune cells, the tumor necrosis factor (TNF) has a significant contribution, which was estimated as a potential target identified for both bioactive compounds of *P. cretica*. The current reverse screening protocol, has revealed many anti-inflammatory targets including Tumor necrosis factor, Estradio 17-beta dehydrogenase-1, cyclooxygenase-2, Interleukin- 4 and VEGFR that have the potential to bind with **CB18** and **F9**, key bioactive compounds of *P. cretica*. 

### 3.4. Neurodegenerative Mechanism

Neurodegeneration being multi-factorial represents a complex pathogenesis. One of the neurodegenerative disorders, Alzheimer’s disease (AD), is characterized by the development of plaques formed by the aggregation of beta-amyloid (Aβ) peptide, responsible for causing neuronal death [78]. Amyloid precursor protein (APP) that undergoes a cleavage by β-secretase, is an attractive target for developing potent inhibitors. Furthermore, secretases that cleave amyloid precursor protein are thought to be potential targets for inhibiting APP cleavage [79]. It has been hypothesized that Aβ protein accumulates inside the brain causing neurons to die. Hence, the inhibition of Aβ aggregation and deposition by inhibiting COX 1 and 2 is considered to play a significant role in treating neuronal damage [80,81]. Neurofibrillary tangles formed as result of Tau protein phosphorylation have been found to contribute towards progression of neurodegeneration through paired helical filament-tau (PHF-tau) production. It has been observed that dephosphorylation caused by kinases: Glycogen synthase kinase-3β (GSK-3β), cyclin-dependent protein kinase 5 (cdk5) and c-Jun N-terminal kinases (JNKs), alleviates cognitive decline [82,83]. Cholinesterases, specifically, acetylcholinesterase (AChE) and butyrylcholinesterase (BuChE) are responsible for the catalytic hydrolysis of important neurotransmitter acetylcholine and butyrylcholine, respectively [84]. Moreover, the inhibition of human carboxylesterase (hCE-1) being similar in structure to cholinesterases (ChE) is also considered as an effective strategy [85]. Oxidative stress has been linked to the neuronal cell death since long. The induction of nitric oxide (NO) produced by nitric oxide synthase (NOS) has been found to play a role in oxidative damage linked to neurodegeneration [86]. Pro-inflammatory cytokines have been found responsible for neuroinflammation leading to neurodegeneration of which TNF-α tops the list [87]. To enhance neuroprotection and cognitive function, the inhibition of phosphodiesterase-5 (PD-5) has been found useful [88]. All above-mentioned targets are perfectly identified by bioactive compounds (**CB18** and **F9**) which estimated the neuroprotective potential of *P. cretica* (Table 1).

### 3.5. Biological Activities of Isolates 

Due to experimental and financial constraints, some of the identified targets were selected to evaluate the potential of bioactive compounds from *P. cretica* against antibacterial and enzymatic inhibition activities. To further estimate the therapeutic potential, other identified targets were taken into account and investigated through molecular modeling studies. 

### 3.6. Antibacterial Activities

The antibacterial activities of two isolated compounds were performed against selected bacteria by applying MABA and results have been reported in Table 2.

For testing of antibacterial activity of isolated compounds, MABA was employed and results have been tabulated in Table 1. The compounds **F9** and **CB18** did not demonstrate any activity against *K. pneumoniae, S. flexneri* and *P. aeruginosa* whilst showed less significant antibacterial activity against *E. coli* (i.e., 11.33% and 14.33%) and *S. aureus* (i.e., 14% and 10%) as compared to ampicillin at 1 mg/mL.

### 3.7. Antioxidant Activities

Purified compounds were subjected to test their antioxidant potential by the DPPH assay and the results have been tabulated in Table 3.

### 3.8. Enzyme Inhibition Activities 

The isolated compounds were also studied for their enzyme inhibition potential against AChE and GluE. The results in terms of percentage inhibition have been mentioned in Table 4 and Table 5.

Table 3 and Table 4 revealed the results of enzyme inhibition activity. At the test concentration (i.e., 0.5 mM) **CB18** depicted slight AChE inhibition activity of 13.25% in comparison to the Eserine standard that showed 91.27% enzyme inhibition. These compounds were also tested for GluE inhibition activity. The compounds **F9** and **CB18** with a concentration of 0.5 mM displayed reasonable 43.82% and 42.35% enzyme inhibition activity, respectively; whereas Acarbose (standard drug) presented 92.23% inhibition.

### 3.9. Molecular Docking and MD Simulations

**Figure 3:**
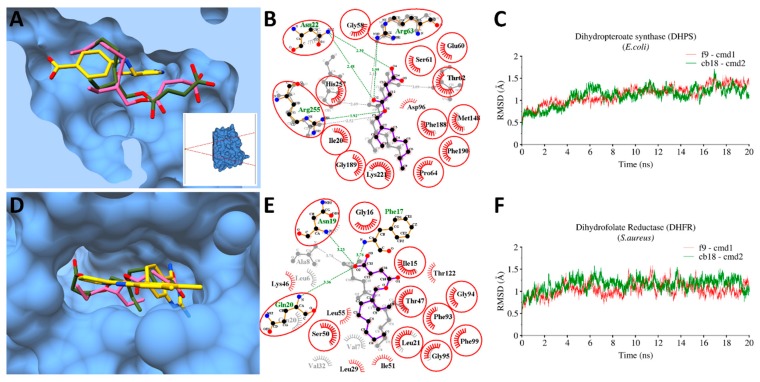
Molecular interactions and molecular dynamics (MD) simulations of CB18 and F9 against *E. coli*-Dihydropteroate synthase (DHPS) and *S. aureus-*Dihydrofolate Reductase (DHFR). (**A**) The molecular surface representation of DHPS with bound pteroic acid (gold), **CB18** (pink) and **F9** (green) superimposed together; (**B**) 2D-interaction plots with *E. coli-*DHPS after MD-simulations, hydrophobic interacting residues are labeled black with spiked red arc, while those interacting through H-bonds are labelled green with distance in angstrom. Conserved residues are marked with red circles; (**C**) root mean square deviation (RMSD) trajectory plots of *E. coli-*DHPS with bound **CB18** (green) and **F9** (red) over a period of 20 ns. The same representations of corresponding *S. aureus-*DHFR with bound **CB18** and **F9** are displayed in (**D**–**F**) in the same color schemes.

**Figure 4:**
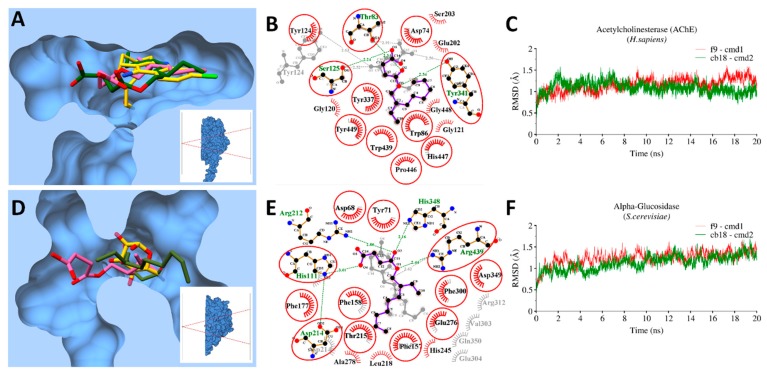
Molecular interactions and MD simulations of **CB18** and **F9** against AChE and *S. cerevisiae*-α-glucosidase. (**A**) The molecular surface representation of acetylcholinesterase (AChE) with bound co-crystalized ligand (gold), **CB18** (green) and **F9** (pink) superimposed together; (**B**) 2D-interaction plots with AChE after MD-simulations, hydrophobic interacting residues are labeled black with spiked red arc, while those interacting through H-bonds are labelled green with distance in angstrom. Conserved residues are marked with red circles; (**C**) RMSD trajectory plots of AChE with bound **CB18** (green) and **F9** (red) over a period of 20 ns. The same representation of corresponding *S. cerevisiae*-α-glucosidase with bound **CB18** and **F9** are displayed in (**D**–**F**) respectively in the same color schemes.

Molecular docking was employed to investigate the binding pose of **CB18** and **F9** with corresponding proteins, which were validated experimentally after reverse docking predictions. Molecular docking revealed fairly good binding affinities against AChE (−6.6 and 6.5 kcal/mol), alpha-glucosidase (−6.9 and 6.2 kacal/mol) and also with bacterial targets, *E. coli-*DHPS (−6 and −5.9 kcal/mol) and *S. aureus-*DHFR (−6.2 and −6.1 kcal/mol), for **CB18** and **F9** respectively. To check the all-atoms backbone stability of proteins in the presence of the corresponding ligand. MD simulations was performed to check the fluctuations in the root-mean-square-deviation (RMSD) of backbone atoms over a time period of 20 ns and analyzed together with co-crystalized ligand of corresponding PDB using 2D-ligplot and chimera molecular surface representation. The overall binding interaction analysis with bacterial enzymatic targets is illustrated in Figure 3 and Figure 4. 

#### 3.9.1. Bacterial Targets 

Overall, the generated RMSD plot for *E. coli-*DHPS and *S. aureus-*DHFR complexed with **CB18** and **F9** were quite stable and remain converged between ~ 0.75 Å. This stability was evident with the stable conformation of the ligand inside the binding pocket during the entire simulation period (Figure 3). The 3D-conformations of **CB18** and **F9** were found deep inside the binding pocket together with the corresponding co-crystalized pteroic acid [57] and [58] in *E. coli-*DHPS and *S. aureus-*DHFR, respectively (Figure 3A,D). 

The 2D interaction analysis after 20 ns revealed the contribution of fumarate moiety in establishing H-bonds (<3 Å distance), while 2-ethylhexyl and 2-ethyloctyl through hydrophobic interactions with the binding site residues of *E. coli-*DHPS and *S. aureus-*DHFR respectively. The fumarate moiety interacted through H-bonds with common residues, including Asn22, Arg63 and Arg255, whereas **F9** established additional H-bonds with Thr62 and His257 (Figure 3B). Likewise, with DHFR, Asn19 and Gln20 were commonly interacted through H-bonds, while the additional H-bond was observed in **F9** and **CB18** with Ala8 and Phe17 residues. Apart from H-bonds, a large number of conserved hydrophobic interactions were also observed including Ile20, Gly58, Glu60, Ser61, Pro64, Met148, Phe188, Gly189, Phe190, and Lys221 in *E. coli*-DHPS, whereas Ile15, Gly16, Leu21, Thr47, Ser50, Phe93, Gly94, Gly95, and Phe99 in *S. aureus-*DHFR.

#### 3.9.2. Enzymatic Targets 

The RMSD trajectory plots of corresponding enzymes with bound ligands showed a similar trend, where the complexes remained converge between 0.75–1.25Å in AChE and *S. cerevisiae*-α-glucosidase. Both compounds were found to have a similar binding pose deep inside the hydrophobic groove of both enzymes when superimposed on reported co-crystalized complexes [55] (Figure 4A,D). Likewise, in the interaction of **CB18** and **F9** with other bacterial targets (Figure 3B,E), fumarate moiety actively contributed in H-bond interactions (<3Å distance) with conserved residues, including Thr83, Ser125 and Tyr341 in AChE, where **F9** established one additional H-bond with Tyr124 (Figure 4B). With α-Glucosidase, His111, Asp214, Arg439 were revealed conserve residues in establishing H-bonds with both ligands whereas **CB18** established additional H-bonds with His348 and Arg212 (Figure 4E). 

### 3.10. MMGBSA Binding Free Energy Calculations

To further explore the binding free energy calculations between selected proteins with **CB18** and **F9** compounds separately, MMGBSA calculations were performed. The MMGBSA total energy (ΔG_tol_) was divided into molecular mechanics (ΔE_MM_) and solvation energy (ΔG_sol_) contributions. ΔE_ele_ is further divided into van der Waals (ΔE_vdw_) and electrostatic energy contributions (ΔE_ele_) while solvation energy is divided into polar (ΔG_p_) and nonpolar (ΔG_np_) contributions. A total of 1000 snapshots were extracted from the whole trajectory for binding free energy calculations. The predicted total binding affinities and contributing energies are tabulated in Table 6. 

The MMGBSA calculations along with molecular mechanics (ΔE_MM_) and solvation energy (ΔG_sol_) contributions are tabulated in Table 6. The estimated MMGBSA values for the targets were slightly consistent with the experimental results. For instance, the MMGBSA binding free energy was very promising with both compounds, **CB18** and **F9** against α-GluE (ΔG_tol_ = −39.86 kcal/mol; ΔG_tol_ = −34.92 kcal/mol), the results were apparent with reasonable 43.82% (**CB18**) and 42.35% (**F9**) enzyme inhibition activity with respect to Acarbose (standard drug) (92.23%) at a concentration of 0.5 mM. While, with antibacterial potential targets, **CB18** and **F9** revealed lower ΔG_tol_ with *E. coli* DHPS (ΔG_tol_ = −18.7 kcal/mol and ΔG_tol_ = −12.79 kcal/mol) and *S. aureus* DHFR (ΔG_tol_ = −17.75 kcal/mol and ΔG_tol_ = −14.73 kcal/mol) which were evident from less significant antibacterial activity of both compounds (**CB18** and **F9**) against *E. coli* (i.e., 11.33% and 14.33%) and *S. aureus* (i.e., 14% and 10%) as compared to ampicillin (90% and 95%) at 1 mg/mL, respectively. Likewise, the estimated ΔG_tol_ of **CB18** and **F9** with AChE also showed higher binding energy (ΔG_tol_ = −28.29 kcal/mol and ΔG_tol_ = −31.52 kcal/mol).

## 4. Conclusions

Through bioassay direction, two maleates were isolated from *P. cretica* and their structures were elucidated. Target potentials were evaluated by reverse docking and biological potentials of the isolates were measured for best-targeted potentials. The results show that the isolates are moderately active against all tested activities. Consequently, it can be said that the isolates have poly-pharmacological potentials. Further study is required to fully explore this plant species. 

## Figures and Tables

**Table 1:** Potential therapeutic targets of isolates **F9** and **CB18** identified by PharmMapper and ReverseScreen3D.

Drug Target Proteins	F9		CB18		Experimental Evidence
	Target Hit	Fit Score	Target Hit	Fit Score	
EGFR	✓	3.48	✓	4.96	PubChem Bioassay
Estrogen receptor beta	n.a	n.a	✓	3.996	NPACT
Cytochrome P450 2C9	✓	4.22	n.a	n.a	NPACT
Acetylcholinesterase (AChE)	✓	4.52	✓	4.3	PubChem Bioassay
TGF-beta receptor type-1	✓	3.75	n.a	n.a	Drug Bank
phosphodiesterase-5 (PD-5)	✓	4.72	n.a	n.a	Drug Bank
Cell dependent kinase 2	n.a	n.a	✓	2.65	PubChem Bioassay
Caspase 3	✓	4.67	✓	5.02	NPACT
Deoxycytidine kinase	✓	2.66	n.a	n.a	Drug Bank
human carboxylesterase (hCE-1)	✓	3.87	n.a	n.a	PubChem Bioassay
Fibroblast growth factor receptor 1, FGFR1	✓	3.41	✓	4.96	PubChem Bioassay
Caspase 7	✓	2.41	n.a	n.a	PubChem Bioassay
STAT-3	✓	4.93	✓	5.39	PubChem Bioassay
Cyclin dependent kinase 6	✓	3.81	✓	4.34	Drug Bank
Cyclin A2	n.a	n.a	✓	3.95	NPACT
p53	✓	4.19	n.a	n.a	Drug Bank
glycogen synthase kinase-3β (GSK-3β)	✓	4.17	✓	3.5	Drug Bank
Apoptosis regulator Bcl-X	✓	4.88	n.a	n.a	PubChem Bioassay
nitric oxide synthase (NOS)	✓	3.39	✓	3.1	PubChem Bioassay
vascular endothelial growth factor, VEGF	✓	3.44	✓	4.18	PubChem Bioassay
MAP kinase 14	✓	3.63	n.a	n.a	NPACT
Histone deacetylase 8	✓	4.26	✓	4.21	PubChem Bioassay
Tumor necrosis factor-alpha	✓	5.34	✓	4.97	PubChem Bioassay
NF-κB	n.a	n.a	✓	2.94	PubChem Bioassay
Prothrombin	n.a	n.a	✓	4.09	Drug Bank
Ornithine aminotransferase, mitochondrial	✓	3.78	✓	2.97	PubChem Bioassay
Sepiapterin reductase	✓	4.81	n.a	n.a	NPACT
VEGFR2	✓	5.11	✓	4.62	PubChem Bioassay
Cyclin dependent kinase 4	n.a	n.a	✓	2.54	Drug Bank
cyclooxygenase-2, COX2	✓	4.14	✓	5.39	Drug Bank
3-phosphoinositide-dependent protein kinase 1	n.a	n.a	✓	4.41	PubChem Bioassay
Beta-secretase 1	✓	3.18	✓	4.39	Drug Bank
JNK1	✓	2.86	✓	3.47	PubChem Bioassay
RAC-alpha serine/threonine-protein kinase	✓	4.55	n.a	n.a	PubChem Bioassay
Estradio 17-beta dehydrogenase-1	✓	3.97	✓	4.58	PubChem Bioassay
Interleukin- 4	✓	2.67	n.a	n.a	PubChem Bioassay

**Table 2:** Antibacterial activities (% inhibition) of isolates from *P. cretica* and standard ampicillin at 1 mg/mL. Each value is average of three repeated experiments ± standard error (S.E.).

Compounds	*E. coli*	*S. aureus*	*K. pneumoniae*	*S. flexneri*	*P. aeruginosa*
**F9**	11.33 ± 0.66	14.00 ± 0.57	-	-	-
**CB18**	14.33 ± 0.33	10.00 ± 0.00	-	-	-
Ampicillin	90.67 ± 0.66	95.00 ± 0.00	80.00 ± 0.00	90.66 ± 0.66	91.33 ± 0.88

**Table 3:** Antioxidant activity of isolated compounds. Each value represents *IC_50_* of average of three repeated experiments ± standard error (S.E.).

Ser. No.	Compounds	DPPH (*IC_50_*) mM
1	F9	02.17 ± 0.49
2	CB18	02.93 ± 0.51
3	Vitamin C	0.029 ± 0.47

All isolates exhibited *IC_50_* in millimolar (mM) range. Their results were not promising as compared to vitamin C. This effect could stem from the absence of any functional groups capable of reduction.

**Table 4:** AChE inhibition activities of isolates (**F9** & **CB18**) at 0.5 mM with Eserine as standard. The values represent average percentage inhibition ± standard error (S.E.) of three repeated experiments.

Ser. No.	Compounds	Inhibition (%) at 0.5 mM AChE
1	**F9**	N.D.
2	**CB18**	13.25 ± 0.14
3	Eserine	91.27 ± 1.17
N.D. = not detected.		

**Table 5:** GluE inhibition activity of isolates (**F9** & **CB18**) at 0.5 mM with Acarbose as standard. Each value represents average percentage inhibition ± standard error (S.E.) of three repeated experiments.

Ser. No.	Compounds	Inhibition (%) at 0.5 mM
1	**F9**	43.82 ± 0.17
2	**CB18**	42.35 ± 0.19
3	Acarbose	92.23 ± 0.16

**Table 6:** Binding affinities and contributing energies of the isolates.

Identified Targets	Bioactive Compounds	ΔE_vdw_	ΔE_ele_	ΔE_MM_	ΔG_p_	ΔG_np_	ΔG_sol_	ΔG_tol_
AChE	**CB18**	−40.05	−8.67	−48.72	23.89	−3.46	20.43	−28.29
	**F9**	−39.98	−9.56	−49.54	22.13	−4.11	18.02	−31.52
Gluco	**CB18**	−45.64	−10.21	−55.85	23.68	−7.69	15.99	−39.86
	**F9**	−43.54	−11.33	−54.87	26.39	−6.44	19.95	−34.92
DHPS (*E. coli*)	**CB18**	−24.2	−8.27	−32.47	16.41	−2.64	13.77	−18.7
	**F9**	−21	−5.61	−26.61	17.03	−3.21	13.82	−12.79
DHFR (*S. aureus*)	**CB18**	−28.44	−5.25	−33.69	20.26	−4.32	15.94	−17.75
	**F9**	−26.21	−4.22	−30.43	21.59	−5.89	15.7	−14.73

## Supplementary Materials

The following are available online at https://www.mdpi.com/2076-3921/8/7/231/s1, Table S1. Detailed absorption, distribution, metabolism, toxicity and excretion in silico assessment.
