# Supplementary material for: Bioassay Directed Isolation, Biological Evaluation and in Silico Studies of New Isolates from Pteris cretica L
Source: Antioxidants (Basel). 2019 Jul 19;8(7):231. doi: 10.3390/antiox8070231 (PMC6680627; doi:10.3390/antiox8070231)
Supplement: Supplementary file 1 [file antioxidants-08-00231-s001.pdf]

Table S1. Detailed absorption, distribution, metabolism, toxicity and excretion in silico assessment.

Attributes	F9	CB18
**Molecular Property**		
Mass	230.304	258.35
logP	2.61	3.39
H-bond acceptors	4	4
H-bond donors	1	1
Rotatable bonds	10	11
PSA	96.98	109.71
Refractivity	62.85	72.46
**Druglikeness**		
Lipinski	Yes; 0 violation	Yes; 0 violation
Ghose filter	Yes	Yes
Veber (GSK) filter	Yes	No; 1 violation: Rot > 10
Egan (Pharmacia) filter	Yes	Yes
Muegge (Bayer) filter	Yes	Yes
Abbott Bioavailability score	0.56	0.56
**Medicinal Chemistry (Friendly)**		
PAINS	0 alert	0 alert
Brenk structural alert	0 alert	0 alert
**ADMET**		
**A (Absorption)**		
Water solubility	-1.913	-2.73
Human Intestinal Absorption (HIA)	95.87	94.5
Human oral bioavailability (HOB)		
Caco-2 permeability	0.92	0.89
P-glycoprotein inhibitior I/II	No	No
P-glycoprotein substrate	No	No
**D (Distribution)**		
BBB Permeability	Yes	Yes
**M (Metabolism)**		
CYP Inhibitors and Substrates		
CYP1A2 inhibition	No	No
CYP2C19 inhibition	No	No
CYP2C9 inhibition	No	No
CYP2C9 substrate	No	No
CYP2D6 inhibition	No	No
CYP2D6 substrate	No	No
CYP3A4 inhibition	No	No
CYP3A4 substrate	No	No
**E (Excretion)**		
Renal Organic Cation Transporter	-	-
**T (Toxicity)**		
AMES toxicity	No	No
hERG I inhibitor	No	No
hERG II inhibitor	No	No
Hepatotoxicty	No	No
Carcinogenicity	No	No
Mutagenic	No	No
Irritant	No	No
